# *Notes from the Field*: *Baylisascaris procyonis* Encephalomyelitis in a Toddler — King County, Washington, 2017

**DOI:** 10.15585/mmwr.mm6702a6

**Published:** 2018-01-19

**Authors:** Vance Kawakami, Amanda Casto, Niranjana Natarajan, Anna Snyder, Jonathan Mosser, Jesse Bonwitt, Matthew P. Kronman, Meagan Kay

**Affiliations:** ^1^Public Health–Seattle & King County, Seattle, Washington; ^2^Epidemic Intelligence Service, Division of Scientific Education and Professional Development, CDC; ^3^University of Washington, Seattle; ^4^University of Washington, Department of Neurology, Seattle; ^5^Seattle Children’s Hospital, Washington; ^6^Washington State Department of Health, Shoreline, Washington.

On May 1, 2017, in Washington, Public Health—Seattle & King County (PHSKC) was notified of a possible *Baylisascaris procyonis* infection in a previously healthy male child aged 19 months. The patient had been evaluated on April 26 for a 1-week history of irritability followed by tremors of his extremities, ataxia, and decreased interactivity. On examination, the patient was afebrile with an inability to sit or stand unaided; complete blood count revealed eosinophilia (absolute eosinophils = 5,080; reference range = 0–250); magnetic resonance imaging (MRI) of the brain indicated diffuse, patchy white matter lesions, alongside patchy, enhancing lesions in both cerebellar hemispheres. The patient was transferred to a tertiary care hospital on April 27 for further evaluation and management; spinal MRI and ophthalmologic exams were normal. Cerebrospinal fluid (CSF) was notable for 4 white blood cells (reference range = 0–5); however, increased eosinophils were noted on cytologic review.

The patient’s parents reported that the child had ingested soil and animal feces in their backyard several weeks before symptom onset. After consultation with CDC, empirical treatment with oral albendazole (25 mg/kg/day) and intravenous steroids for suspected baylisascariasis was initiated on April 29, while *B. procyonis* antibody test results were pending. The patient exhibited neurologic improvement on May 1 and was discharged on May 2.

Serum and CSF specimens collected during hospitalization were positive for *B. procyonis*–specific immunoglobulin antibodies at CDC; results of Toxocara and Toxoplasma serologies and a stool ova and parasite exam conducted at private laboratories were negative. The assay for *B. procyonis* antibody, an immunoblot test using the recombinant antigen rBpRAG1, is specific and sensitive but cannot differentiate between current or previous infection or exposure (*1*). The patient completed 28 days of albendazole and a steroid taper. At 1-month follow up, the patient had marked reduction in tremors and improvements in mental status and ambulation.

*B. procyonis* is a roundworm parasite of the North American raccoon (*Procyon lotor*), the definitive host. Infection with *B. procyonis* is a rare cause of morbidity and mortality in humans, with 31 cases documented in North America (*2*,*3*). Infection occurs when humans ingest infective egg stages shed in raccoon feces or material contaminated with raccoon feces (*2*). Clinical signs of baylisascariasis depend on the dose of ingested eggs and their extraintestinal migration path (neural, ocular, or visceral tissue). Among the 31 documented North American cases of disease, 28 (90%) persons had meningoencephalitis or encephalopathy (*2*,*3*).

PHSKC conducted an environmental assessment of the patient’s property on May 9. Dark, cylindrical feces were collected from elevated truncal forks (approximately 8.2 ft [2.5 m] and 13.1 ft [4 m] in height) and base of the tree at the site where the patient regularly played and was seen ingesting soil and animal feces. The fecal characteristics and location at multiple sites were consistent with a raccoon latrine (i.e., communal defecation site) (*4*). A fecal sample was also collected from the patient’s healthy dog.

All fecal samples collected from the tree yielded microscopic eggs consistent in morphology and size to *B. procyonis*. No parasite ova were detected in the fecal sample collected from the dog. The patient’s parents had not previously noticed any raccoon latrines on their property. PHSKC recommended restricting access to the tree and surrounding areas until it could be appropriately cleaned and consulting with a veterinarian about implementing regular deworming for their dog, because canines can be a definitive host for *B. procyonis* and can shed eggs in their feces (*3*).

This report describes the first laboratory-confirmed *B. procyonis* human infection in Washington. Children aged <2 years exhibit frequent hand-to-mouth behaviors and might be at increased risk for baylisascariasis through ingestion of soil and other potentially contaminated material (e.g., contents of sandboxes) (*2*). Among the 31 documented disease cases, 17 (55%) were among children aged <2 years (*2*,*3*). Prevention messages to parents of young children living in areas where raccoons might be present should include avoidance of soil ingestion, handwashing after outdoor play, and providing guidance on identifying and safely cleaning raccoon latrines (*4*).

## References

[1] Rascoe LN, Santamaria C, Handali S, Interlaboratory optimization and evaluation of a serological assay for diagnosis of human baylisascariasis. Clin Vaccine Immunol 2013;20:1758–63. 10.1128/CVI.00387-13 24049107 PMC3837780

[2] Graeff-Teixeira C, Morassutti AL, Kazacos KR. Update on baylisascariasis, a highly pathogenic zoonotic infection. Clin Microbiol Rev 2016;29:375–99. 10.1128/CMR.00044-15 26960940 PMC4786883

[3] Sircar AD, Abanyie F, Blumberg D, Raccoon roundworm infection associated with central nervous system disease and ocular disease—six states, 2013–2015. MMWR Morb Mortal Wkly Rep 2016;65:930–3. 10.15585/mmwr.mm6535a2 27608169

[4] CDC. Raccoon latrines: identification and clean-up. Atlanta, GA: US Department of Health and Human Services, CDC; 2016. https://www.cdc.gov/parasites/baylisascaris/resources/raccoonlatrines.pdf

